# The New Parliament

**Published:** 1910-12-17

**Authors:** 


### The New Parliament.

The under-representation of medicine in the House of Commons is a familiar text for lamentation in the medical Press on behalf both of that assembly itself, of the profession as well, and of the nation at large. The Parliament whose election is now almost completed differs but little from its predecessors in this respect. There are, it is true, several members who possess medical qualifications, but most of them abandoned long ago the practice of medicine and entered upon other careers which have been the means of their advancement to the estate of legislators. Of such one-time doctors familiar examples are Sir R. B. Finlay, Sir G. S. Robertson, Mr. Dillon, Dr. A. R. Rainy, and Mr. McCurdy. The reason for this state of affairs is quite clear. The long and severe curriculum demanded of the medical student who would obtain admission to the Register effectually deters those from entering it who have no need or desire to practise, but intend to enter politics after their ordinary liberal education. Such fortunate persons prefer the Bar, if they think of qualifying as members of a profession at all; for to be called is both easier to attain and perhaps more likely to be of service than to study medicine. The Army, the Diplomatic Service, and similar careers also find occupation for the early manhood of those whose ultimate object is to become the rulers of their fellow-countrymen. Thus it is that few of those who enter the House of Commons before middle age have had the benefits of a medical training.

Turning to those whose ambition leads them to solicit the favour of a constituency when their active careers in their respective walks of life are over, it is easy to understand why so few doctors find their way to St. Stephen's. A medical practice is essentially a personal thing; it cannot be carried on by deputy, nor can the owner of it consult his own convenience as to what shall be his working hours. It is out of the question for any general practitioner to go into Parliament unless he is prepared to give up active practice. Therefore, apart from consultants such as Sir Wm. Collins and Sir Victor Horsley (two of the unsuccessful medical candidates at this election), and teachers such as Dr. Addison, it is really only retired doctors who can aspire to Parliamentary life. To retire from practice means to give up all income derived from one's profession; and the number of medical men who can afford to do this, save at an age when leisure is more to be desired than being heckled, is extremely small, as we all know.

So far, the story is a very old one. Is there any prospect of a remedy? It is known that the party in power intends to introduce at the earliest opportunity payment of members and relief from much of the expense that now attaches to candidature. It will be strange if this system, should it be brought into action, does not enable medical men to enter the House more freely than they have hitherto been able to do. Whatever other results may ensue from the measure, at least from this point of view a clear gain is in prospect; for vital questions of public health, hygiene, eugenics, and other matters, unsurpassed in importance by any with which the House of Commons has to deal, will then receive far more attention and far better handling than is possible at present in an assembly consisting mainly of lawyers, soldiers, miners, and millionaires of mixed extraction. Amid the uproar of zealous partisan warfare the affairs of the nation receive but too often scant notice, and questions of the national welfare which involve medical points are the first to suffer as a rule. It would seem too much to hope that in 1911 the strife of contending factions will be any less absorbing than in 1910, devoutly though it were to be wished for the sake of everybody but the professional politicians. But the little band of doctors in Parliament must see to it that the opinion of the profession on matters of sociological medicine is voiced in a manner that will secure the ear of the House. From a strictly nonparty aspect, the defeat of Sir W. J. Collins at the polls is so serious a loss that it behoves the others all the more to bear in mind their responsibilities.

